# Tænia

**Published:** 1855-01

**Authors:** 


Tænia.—When the usual remedies for the expulsion of tapeworm fail, try tatzé and saoria.—*Ibid.*

